# Supplementary material for: Smartphone Photogrammetric Assessment for Head Measurements
Source: Sensors (Basel). 2023 Nov 6;23(21):9008. doi: 10.3390/s23219008 (PMC10648760; doi:10.3390/s23219008)
Supplement: Supplementary file 1 [file sensors-23-09008-s001.zip › sensors-2669489-supplementary.pdf]

sensors

MDPI

Supplementary Materials

Accuracy Assessment Smartphone Galaxy S22. The degree repeatability of the distances obtained for the 12 PhotoMeDAS models obtained with the Galaxy S22 smartphone for: **Processing I** , **Processing II** and **Processing III**, can be analysed first by observing the differences in Tables S1.1, S1.2 and S1.3.

Table S1.1. Results Processing I with Samsung Galaxy S22.

Model	Dist. Preauricular (mm)	Dist. Lateral (mm)	Dist. Max right (mm)	Dist. Max left (mm)	Variation Preauricular (mm)	Variation Lateral (mm)	Variation Max right (mm)	Variation Max left (mm)
1	128.8	141.0	171.9	170.5	-1.6	-0.8	-1.1	-1.0
2	128.1	140.9	172.1	170.8	-1.0	-0.7	-1.3	-1.3
3	128.0	141.1	172.4	171.1	-0.8	-1.0	-1.6	-1.7
4	129.0	141.8	172.6	171.4	-1.8	-1.6	-1.8	-1.9
5	127.6	140.3	172.1	170.7	-0.4	-0.1	-1.3	-1.1
6	129.4	141.6	172.4	171.1	-2.1	-1.4	-1.6	-1.7
7	128.1	141.3	172.3	171.0	-1.0	-1.1	-1.5	-1.5
8	127.4	141.1	172.4	171.1	-0.1	-0.9	-1.6	-1.6
9	127.8	140.4	172.0	170.8	-0.6	-0.1	-1.1	-1.3
10	127.7	140.8	171.7	170.3	-0.5	-0.6	-0.9	-0.8
11	128.3	140.8	171.7	170.3	-1.1	-0.6	-0.9	-0.8
12	128.1	141.9	173.3	171.8	-1.0	-1.7	-2.5	-2.3
**Minimum**	127.4	140.3	171.7	170.3	-2.1	-1.7	-2.5	-2.3
**Maximum**	129.4	141.9	173.3	171.8	-0.1	-0.1	-0.9	-0.8
**Range**	2.0	1.6	1.6	1.5	2.0	1.6	1.6	1.5
**Average**	**128.2**	**141.1**	**172.2**	**170.9**	**-1.0**	**-0.9**	**-1.4**	**-1.4**
**Std. deviation**	0.6	0.5	0.4	0.4	0.6	0.5	0.4	0.4

Table S1.2. Results Processing II with Samsung Galaxy S22.

Model	Dist. Preauricular (mm)	Dist. Lateral (mm)	Dist. Max right (mm)	Dist. Max left (mm)	Variation Preauricular (mm)	Variation Lateral (mm)	Variation Max right (mm)	Variation Max left (mm)
1	128.3	141.3	171.7	170.3	-1.1	-0.8	-1.1	-0.9
2	127.6	141.1	172.0	170.6	-0.4	-1.1	-0.9	-1.2
3	128.1	141.6	172.4	171.1	-1.0	-1.7	-1.4	-1.6
4	128.9	142.1	172.4	171.1	-1.7	-1.6	-1.9	-1.6
5	127.4	140.7	172.1	170.4	-0.2	-0.9	-0.5	-1.3
6	129.1	141.9	172.1	170.9	-2.0	-1.4	-1.7	-1.3
7	127.7	141.3	171.7	170.5	-0.5	-1.0	-1.1	-0.9
8	127.1	141.1	172.1	170.9	0.1	-1.4	-1.0	-1.4
9	128.0	141.4	171.1	170.1	-0.8	-0.6	-1.2	-0.4
10	127.9	141.1	171.5	170.1	-0.7	-0.6	-1.0	-0.7
11	128.3	141.1	171.6	170.1	-1.1	-0.7	-1.0	-0.8
12	128.5	142.1	173.1	171.8	-1.3	-2.3	-2.0	-2.4
**Minimum**	127.1	140.7	171.1	170.1	-2.0	-2.3	-2.0	-2.4
**Maximum**	129.1	142.1	173.1	171.8	0.1	-0.6	-0.5	-0.4
**Range**	2.0	1.4	2.0	1.7	2.1	1.7	1.5	2.0
**Average**	**128.1**	**141.4**	**172.0**	**170.7**	**-0.9**	**-1.2**	**-1.2**	**-1.2**
**Std. deviation**	0.6	0.4	0.5	0.5	0.6	0.5	0.4	0.5

Table S1.3. Results Processing III with Samsung Galaxy S22.

Model	Dist. Preauricular (mm)	Dist. Lateral (mm)	Dist. Max right (mm)	Dist. Max left (mm)	Variation Preauricular (mm)	Variation Lateral (mm)	Variation Max right (mm)	Variation Max left (mm)
1	127.8	141.1	171.6	170.3	-0.6	-0.9	-0.8	-0.8
2	127.7	141.1	171.6	170.1	-0.5	-0.9	-0.8	-0.6
3	128.1	141.5	172.5	171.1	-0.9	-1.3	-1.7	-1.7
4	128.1	141.8	173.0	171.6	-1.0	-1.6	-2.2	-2.1
5	127.7	140.7	172.1	170.7	-0.5	-0.5	-1.4	-1.2
6	128.5	141.7	172.4	171.1	-1.3	-1.5	-1.6	-1.6
7	128.0	141.4	172.0	171.0	-0.8	-1.2	-1.2	-1.5
8	127.1	141.1	171.9	170.6	0.0	-1.0	-1.1	-1.1
9	127.5	141.0	171.3	170.1	-0.3	-0.8	-0.5	-0.6
10	126.9	140.6	171.4	170.1	0.3	-0.4	-0.6	-0.6
11	128.1	141.0	171.6	170.4	-0.9	-0.8	-0.8	-0.9
12	128.9	142.4	173.1	171.8	-1.7	-2.2	-2.3	-2.3
**Minimum**	126.9	140.6	171.3	170.1	-1.7	-2.2	-2.3	-2.3
**Maximum**	128.9	142.4	173.1	171.8	0.3	-0.4	-0.5	-0.6
**Range**	2.0	1.8	1.8	1.7	2.0	1.8	1.8	1.7
**Average**	**127.9**	**141.3**	**172.0**	**170.7**	**-0.7**	**-1.1**	**-1.2**	**-1.3**
**Std. deviation**	0.6	0.5	0.6	0.6	0.5	0.5	0.6	0.6

Accuracy Assessment Smartphone Galaxy S22+. The degree of repeatability of the distances obtained for the 12 PhotoMeDAS models obtained with the Galaxy S22+ smartphone for: **Processing I** , **Processing II** and **Processing III**, can be analysed first by observing the differences in Tables S2.1, S2.2 and S2.3.

Table S2.1. Results Processing I with Samsung Galaxy S22+.

Model	Dist. Preauricular (mm)	Dist. Lateral (mm)	Dist. Max right (mm)	Dist. Max left (mm)	Variation Preauricular (mm)	Variation Lateral (mm)	Variation Max right (mm)	Variation Max left (mm)
13	126.7	139.4	169.0	168.2	0.5	0.8	1.8	1.3
14	126.8	139.3	169.2	168.2	0.4	0.9	1.6	1.3
15	126.8	139.3	169.6	169.0	0.4	0.9	1.2	0.5
16	126.9	139.2	169.5	168.8	0.3	1.0	1.3	0.7
17	127.4	140.0	169.4	168.5	-0.2	0.2	1.4	1.0
18	125.9	139.0	168.8	168.0	1.3	1.2	2.0	1.5
19	127.2	139.3	169.2	168.5	0.0	0.9	1.6	1.0
20	127.1	139.6	169.8	168.9	0.1	0.6	1.0	0.6
21	127.1	139.6	169.6	168.9	0.1	0.6	1.2	0.6
22	126.3	139.3	169.0	168.3	0.9	0.9	1.8	1.2
23	126.8	139.2	169.7	168.4	0.4	1.0	1.1	1.1
24	126.1	138.6	168.4	167.7	1.1	1.6	2.4	1.8
**Minimum**	125.9	138.6	168.4	167.7	-0.2	0.2	1.0	0.5
**Maximum**	127.4	140.0	169.8	169.0	1.3	1.6	2.4	1.8
**Range**	1.5	1.4	1.4	1.3	1.5	1.4	1.4	1.3
**Average**	**126.8**	**139.3**	**169.3**	**168.5**	**0.4**	**0.9**	**1.6**	**1.0**
**Std. deviation**	0.5	0.3	0.4	0.4	0.5	0.3	0.4	0.4

Table S2.2. Results Processing II with Samsung Galaxy S22+.

**Model**	**Dist. Preauricular (mm)**	**Dist. Lateral (mm)**	**Dist. Max right (mm)**	**Dist. Max left (mm)**	**Variation Preauricular (mm)**	**Variation Lateral (mm)**	**Variation Max right (mm)**	**Variation Max left (mm)**
13	126.9	139.7	169.2	168.4	0.3	0.5	1.6	1.1
14	127.1	139.8	169.1	168.3	0.1	0.4	1.7	1.2
15	126.6	139.5	169.4	168.7	0.6	0.7	1.4	0.8
16	127.5	139.9	169.5	168.7	-0.3	0.3	1.3	0.8
17	127.4	140.0	169.5	168.6	-0.2	0.2	1.3	0.9
18	126.8	139.6	168.7	167.9	0.4	0.6	2.1	1.6
19	127.3	139.5	168.9	168.3	-0.1	0.7	1.9	1.2
20	127.2	139.8	169.7	169.0	0.0	0.4	1.1	0.5
21	127.2	139.9	169.5	168.8	0.0	0.3	1.3	0.7
22	126.8	139.9	169.2	168.4	0.4	0.3	1.6	1.1
23	127.2	139.9	169.2	168.1	0.0	0.3	1.6	1.4
24	126.5	139.3	168.5	167.7	0.7	0.9	2.3	1.8
**Minimum**	126.5	139.3	168.5	167.7	-0.3	0.2	1.1	0.5
**Maximum**	127.5	140.0	169.7	169.0	0.7	0.9	2.3	1.8
**Range**	1.0	0.7	1.2	1.3	1.0	0.7	1.2	1.3
**Average**	**127.0**	**139.7**	**169.2**	**168.4**	**0.1**	**0.5**	**1.6**	**1.1**
**Std. deviation**	0.3	0.2	0.4	0.4	0.3	0.2	0.4	0.4

Table S2.3. Results Processing III with Samsung Galaxy S22+.

**Model**	**Dist. Preauricular (mm)**	**Dist. Lateral (mm)**	**Dist. Max right (mm)**	**Dist. Max left (mm)**	**Variation Preauricular (mm)**	**Variation Lateral (mm)**	**Variation Max right (mm)**	**Variation Max left (mm)**
13	126.7	139.7	168.6	167.9	0.5	0.5	2.2	1.6
14	126.5	139.7	169.0	168.1	0.7	0.5	1.8	1.4
15	126.7	139.9	169.4	168.6	0.5	0.3	1.4	0.9
16	126.1	139.7	169.0	168.2	1.1	0.5	1.8	1.3
17	126.8	139.8	169.4	168.7	0.4	0.4	1.4	0.8
18	126.2	139.3	168.4	167.7	1.0	0.9	2.4	1.8
19	126.4	139.2	168.7	168.2	0.8	1.0	2.1	1.3
20	127.4	140.4	169.2	168.7	-0.2	-0.2	1.6	0.8
21	126.7	139.7	169.3	168.7	0.5	0.5	1.5	0.8
22	125.5	139.3	168.7	168.1	1.7	0.9	2.1	1.4
23	127.0	139.8	169.2	167.9	0.2	0.4	1.6	1.6
24	126.5	139.3	168.5	167.7	0.7	0.9	2.3	1.8
Minimum	125.5	139.2	168.4	167.7	-0.2	-0.2	1.4	0.8
Maximum	127.4	140.4	169.4	168.7	1.7	1.0	2.4	1.8
Range	1.9	1.2	1.0	1.0	1.9	1.2	1.0	1.0
Average	**126.5**	**139.7**	**169.0**	**168.2**	**0.6**	**0.5**	**1.9**	**1.3**
Std. deviation	0.5	0.3	0.4	0.4	0.5	0.3	0.4	0.4

Accuracy Assessment Smartphone Galaxy S22 Ultra. The degree of repeatability of the distances obtained for the 12 PhotoMeDAS models obtained with the Galaxy S22 Ultra smartphone for: **Processing I , Processing II** and **Processing III**, can be analysed first by observing the differences in Tables S3.1, S3.2 and S3.3.

Table S3.1 Results Processing I with Samsung Galaxy S22 Ultra.

Model	Dist. Preauricular (mm)	Dist. Lateral (mm)	Dist. Max right (mm)	Dist. Max left (mm)	Variation Preauricular (mm)	Variation Lateral (mm)	Variation Max right (mm)	Variation Max left (mm)
25	128.8	141.8	172.5	171.3	-1.6	-1.6	-1.7	-1.8
26	128.8	142.3	172.7	171.6	-1.6	-2.1	-1.9	-2.1
27	129.3	142.1	172.3	171.4	-2.1	-2.0	-1.5	-1.9
28	129.7	142.1	172.4	171.4	-2.5	-2.0	-1.6	-1.9
29	128.8	141.8	172.5	171.6	-1.6	-1.6	-1.7	-2.1
30	129.7	142.5	172.7	171.8	-2.5	-2.3	-1.9	-2.3
31	128.9	141.8	172.6	171.5	-1.7	-1.6	-1.8	-2.0
32	129.1	142.0	172.6	171.5	-1.9	-1.8	-1.8	-2.0
33	128.9	141.9	172.9	171.8	-1.7	-1.7	-2.1	-2.3
34	128.7	141.7	172.7	171.7	-1.5	-1.5	-1.9	-2.1
35	129.6	142.0	172.1	171.0	-2.4	-1.8	-1.4	-1.5
36	129.4	141.7	172.0	171.0	-2.1	-1.5	-1.1	-1.5
**Minimum**	128.7	141.7	172.0	171.0	-2.5	-2.3	-2.1	-2.3
**Maximum**	129.7	142.5	172.9	171.8	-1.5	-1.5	-1.1	-1.5
**Range**	1.0	0.8	0.9	0.8	1.0	0.8	0.9	0.8
**Average**	**129.1**	**142.0**	**172.5**	**171.5**	**-2.0**	**-1.8**	**-1.7**	**-2.0**
**Std. deviation**	0.4	0.2	0.3	0.3	0.4	0.3	0.3	0.3

Table S3.2. Results Processing II with Samsung Galaxy S22 Ultra.

Model	Dist. Preauricular (mm)	Dist. Lateral (mm)	Dist. Max right (mm)	Dist. Max left (mm)	Variation Preauricular (mm)	Variation Lateral (mm)	Variation Max right (mm)	Variation Max left (mm)
25	128.1	141.4	172.3	171.1	-0.9	-1.1	-1.5	-1.7
26	128.5	142.0	172.5	171.6	-1.3	-1.8	-1.7	-2.1
27	129.1	141.9	172.7	171.8	-2.0	-1.7	-1.9	-2.3
28	129.4	142.1	172.4	171.5	-2.1	-2.0	-1.6	-2.0
29	128.1	141.4	172.4	171.8	-1.0	-1.1	-1.6	-2.3
30	129.8	142.3	173.0	171.9	-2.6	-2.1	-2.1	-2.4
31	129.4	142.1	172.5	171.5	-2.1	-1.9	-1.7	-2.0
32	128.8	141.7	172.4	171.5	-1.6	-1.5	-1.6	-2.0
33	128.4	141.8	172.6	171.7	-1.1	-1.6	-1.8	-2.1
34	128.3	141.5	172.1	171.1	-1.1	-1.3	-1.3	-1.7
35	128.9	141.4	172.3	171.1	-1.7	-1.1	-1.5	-1.7
36	128.6	141.1	172.0	171.1	-1.4	-1.0	-1.1	-1.7
**Minimum**	128.7	141.7	172.0	171.0	-2.6	-2.1	-2.1	-2.4
**Maximum**	129.7	142.5	172.9	171.8	-0.9	-1.0	-1.1	-1.7
**Range**	1.0	0.8	0.9	0.8	1.7	1.1	1.0	0.7
**Average**	**129.1**	**142.0**	**172.5**	**171.5**	**-1.6**	**-1.6**	**-1.6**	**-2.0**
**Std. deviation**	0.4	0.2	0.3	0.3	0.5	0.4	0.3	0.3

Table S3.3. Results Processing III with Samsung Galaxy S22 Ultra.

**Model**	**Dist. Preauricular (mm)**	**Dist. Lateral (mm)**	**Dist. Max right (mm)**	**Dist. Max left (mm)**	**Variation Preauricular (mm)**	**Variation Lateral (mm)**	**Variation Max right (mm)**	**Variation Max left (mm)**
25	127.7	141.1	172.7	171.5	-0.5	-0.9	-1.9	-2.0
26	127.9	141.5	173.5	172.1	-0.7	-1.3	-2.7	-2.7
27	128.7	141.3	173.1	172.0	-1.5	-1.1	-2.3	-2.5
28	129.0	141.8	173.3	172.4	-1.8	-1.6	-2.5	-2.9
29	129.5	142.1	172.6	171.8	-2.3	-2.0	-1.8	-2.3
30	128.7	142.1	173.1	172.3	-1.5	-2.0	-2.3	-2.8
31	130.0	142.5	172.4	171.5	-2.8	-2.3	-1.6	-2.0
32	129.1	142.0	172.4	171.5	-2.0	-1.8	-1.6	-2.0
33	130.3	142.7	173.4	172.4	-3.1	-2.5	-2.6	-2.9
34	128.0	141.4	172.5	171.5	-0.8	-1.1	-1.7	-2.0
35	129.1	141.7	173.0	172.0	-2.0	-1.5	-2.1	-2.5
36	128.6	141.4	172.5	171.1	-1.4	-1.1	-1.7	-1.7
**Minimum**	127.7	141.1	172.4	171.1	-3.1	-2.5	-2.7	-2.9
**Maximum**	130.3	142.7	173.5	172.4	-0.5	-0.9	-1.6	-1.7
**Range**	2.6	1.6	1.1	1.3	2.6	1.6	1.1	1.1
**Average**	**128.9**	**141.8**	**172.9**	**171.8**	**-1.7**	**-1.6**	**-2.0**	**-2.4**
**Std. deviation**	0.8	0.5	0.4	0.4	0.8	0.5	0.4	0.4
